# Learning to drive: resident physicians’ perceptions of how attending physicians promote and undermine autonomy

**DOI:** 10.1186/s12909-019-1732-6

**Published:** 2019-07-31

**Authors:** Cameron Crockett, Charuta Joshi, Marcy Rosenbaum, Manish Suneja

**Affiliations:** 1 Department of Pediatrics, Saint Louis Children’s Hospital, Saint Louis, MO USA; 2 Pediatric Epilepsy Team, Regional Neurology Services, Department of Child Neurology, Children’s Hospital Colorado, Denver, CO USA; 3 Department of Family Medicine, University of Iowa Carver College of Medicine, Iowa City, Iowa, USA; 4 Department of Internal Medicine, University of Iowa Hospitals and Clinics, University of Iowa Carver College of Medicine, Iowa City, IA 52242 USA

**Keywords:** Autonomy, Clinical supervision, Graduate medical education, Resident perceptions

## Abstract

**Background:**

Providing appropriate levels of autonomy to resident physicians is an important facet of graduate medical education, allowing learners to progress toward the ultimate goal of independent practice. While studies have identified the importance of autonomy to the development of resident physicians, less is known about resident perspectives on their “lived experiences” with autonomy and ways in which clinical educators either promote or undermine it. The current study aims to provide an empirically based practical framework based on resident perspectives through which supervising physicians can attempt to more adequately foster resident physician autonomy.

**Methods:**

Residents completed open ended surveys followed by facilitated group discussions of their perspectives on autonomy. Qualitative thematic analysis identified key themes in resident definitions of autonomy and how clinical educators either promote or undermine resident autonomy during supervision. Fifty-nine resident physicians representing six different specialties from two institutions participated.

**Results:**

Learners felt that autonomy was critical to their development as independent physicians. Leading the approach to care, a sense of ownership for patients, and receiving appropriate levels of supervision were identified as key components of autonomy. Attending physicians who promoted this active involvement with patient care were felt to have a strong positive influence on resident autonomy. Autonomy was undermined when decisions were micromanaged and resident input in decision-making process was minimized.

**Conclusions:**

Fostering autonomy is a critical aspect of medical education. Allowing residents to take the lead in the delivery of patient care while supporting them as important members of the health care team can help to promote resident autonomy in the clinical setting.

## Background

While allowing resident physicians (RPs) to act autonomously and learn to practice independently is a critical component of RP education, attending physicians (APs) often struggle with when and how to entrust their RPs with autonomous activities [1]. The concept of graduated level of independence and responsibility form the basis of an initiative in medical education known as competency-based training [2]. During training, supervision is critical in ensuring patient safety, yet adult learning theory highlights that learning occurs when trainees are challenged to work beyond their comfort level [3]. Concerns regarding excessive oversight by faculty, the interplay of trust and graduated levels of independence, the importance of inclusion of RPs in patient care decisions have been addressed in several recent commentaries related to RP autonomy [4–7]. While commentaries provide insight into both RP and AP viewpoints on these issues, and on how best to encourage autonomy through educational practices, empiric investigation into these issues has been limited.

There is an inherent tension between learner autonomy and supervision in clinical training. Multiple theoretical constructs, including self-determination (the inherent tendency for a learner to develop self-directed and autonomous behaviors driven by intrinsic motivation) and scaffolding (how teachers can facilitate learner progress by providing developmental stepwise instructional support) have been used to describe the path of a learner towards an independent practitioner [8]. Competency based medical education (CBME) with focus on milestones and Entrustable Professional Activities (EPA) has allowed us to use these constructs in resident assessment and to optimize the balance between supervision and autonomy [1, 2, 9, 10]. While the concept of graduated level of independence /autonomy based on achievement of milestones and the resident’s ability to perform specific tasks is now better defined in the literature, less is known about resident perspectives and “lived experience” of how autonomy is perceived by the residents and how attendings promote or undermine it [5–10]. Furthermore, most studies investigating learners’ perceptions of training and raising questions regarding autonomy have been based in one specialty area and thus limited in their generalizability across medical disciplines [11, 12]. To address these gaps in the literature, the current study examines ways in which RPs from a variety of specialties feel APs either promote or undermine their autonomous behavior. By detailing a cohesive picture of autonomy as it is perceived by RPs, we aim to provide an empirically based practical framework for fostering RP autonomy.

## Methods

When assessing behavior trends and experiences, particularly about which little is known, it is common to use qualitative methods such as focus groups and open ended surveys [13, 14]. To explore RP’s perspectives on autonomy, we chose to use a qualitative approach guided by phenomenology in order to explore the range of “lived” experiences and meanings attached to these experiences by RPs [15]. This study analyzed RP perceptions of the meaning of autonomy and how APs either promoted or undermined this autonomy.

### Participants

Eight different resident groups met between March and July 2013. We used a convenience sample of RPs representing 6 different specialties at two hospitals. Rather than mixing specialties during these meetings, which may have inhibited candid discussion among people unfamiliar with one another, each specialty group participated in a separate meeting. These homogenous specialty groups were able to discuss commonly held perspectives on autonomy and attending physicians’ behaviors within their own learning context. Program coordinators in each program helped identify convenient meeting times for resident meetings and forwarded informational recruitment emails to all residents in each program asking for their voluntary participation. Program coordinators were not present for any of the sessions. Meetings were conducted during regular RP departmental conferences lasted 45–60 min. This research was approved by the University of Iowa Institutional Review Board.

### Data collection

During the meetings, we used two different methods for collecting resident perspectives on autonomy. Participants first completed written, open-ended questionnaires on RP autonomy (Table 1) that had been initially piloted and refined with input from 3 resident volunteers. This gave meeting participants a chance to reflect on their own experiences and perspectives and allowed us to capture narrative responses from every participant, which is at times difficult to accomplish in group discussions. RPs were asked to define their perception of “autonomy” and to detail how autonomous behavior might be objectively observed in their daily activities, and AP actions that were felt to promote or undermine autonomy. After completing the surveys, facilitated group discussions were conducted by a student researcher (CC) who was trained in focus groups methods by another author (MR), who had extensive training in qualitative interview methods. Responses to written questions were used to loosely guide the flow of these discussions, but open-ended responses and further discussion was encouraged. Our choice to follow individual completion of open ended survey with facilitated group discussion was to allow opportunity for participants to compare their perspectives with each other and build upon and clarify these individual perspectives in their discussion. The interviewers pursued relevant themes and sought clarification as necessary. Written notes and audio recordings of the group discussions allowed reproducibility of the dialogue.

**Table 1:** Resident autonomy questionnaire items. All residents were provided a written questionnaire prior to facilitated group discussions to acquire feedback from all participants and provide a basis for discussion topics

Year in Residency: _________________ Program: _________________________	
1. How do you define “resident autonomy” in patient care?	
2. If someone were to observe you for a day, what would be examples of autonomous behavior that this individual might notice?	
3. What kinds of things do attending physicians do that promote autonomy?	
4. What kinds of things do attending physicians do that undermine autonomy?	
5. Do you have any additional comments on autonomy in resident education?	

### Analysis

**Fig. 1:**
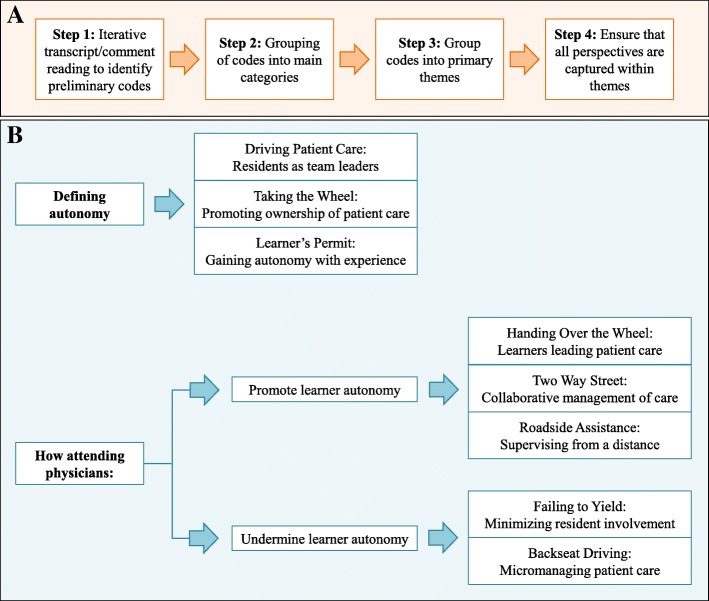
Thematic analysis process identifying resident physician perspectives on autonomy. **a** General approach to coding data using the “editing style” approach; **b** Analysis of data identified several themes which were salient across specialties

Thematic analysis identified salient themes in participants’ perceptions of the meaning and examples of autonomy and ways in which AP actions affected autonomy [13, 14]. All interviews and open-ended questionnaire responses were transcribed verbatim. Two of the authors (CC and MR) independently read through all the survey comments and group discussion transcripts using the “editing style” approach [16] to identify initial themes which provided the basis for a preliminary coding scheme. Each author then applied the coding scheme to a sample of comments and transcripts and compared their coding to resolve any conflicting interpretations or new codes that emerged from the analysis. The final coding scheme was subsequently applied to all the data from the surveys and group conversations using NVivo 8 software, which allowed for systematic searching and sorting of data [17]. To ensure quality and rigor of the data, all coded data and codes were subsequently reviewed by the third author (MS) to ensure that all data could be accounted for by the main themes. See Fig. 1 for a summary of the analysis process and main themes.

## Results

A total of 59 RPs participated in one of eight meetings. All participants completed the open ended surveys and participated in the subsequent group discussions (see Table 2 for discipline and PGY level). Before July 1 (when a new group of interns was transitioned in) groups consisted of RPs in all years of training and after July 1 groups excluded new interns. Many of the comments and phrases used by the study participants pointed to a recurrent analogy comparing autonomy with aspects of driving a motor vehicle, and we based our themes upon this language (Table 3). From these themes we developed a model of RP autonomy (Fig. 1).

**Table 2:** Number and PGY year of resident participants in data collection meetings based on program

Program	PGY-1	PGY-2	PGY-3	PGY-4	PGY-5	Total
Emergency Medicine (EM)	3	4	1	0	0	8
Family Medicine (FM)	0	4	2	0	0	6
Internal Medicine (IM)	0	0	3	0	0	3
Internal Medicine*	0	3	3	0	0	6
Pediatrics	2	6	1	0	0	9
Pediatrics*	0	1	3	0	0	4
Psychiatry	2	2	2	1	0	7
Radiology	0	0	5	4	7	16
All Participants	7	20	20	5	7	59**

Programs marked with an asterisk (*) are community-based programs, all others were programs associated with a major University. One PGY-2 RP from the Psychiatry focus group was a dual Psychiatry/Family Medicine trainee

** All residents completed questionnaires and participated in facilitated group discussion

**Table 3:** Salient themes in analysis of resident physicians’ perspectives on Autonomy

Theme	Components
Defining Resident Physician Autonomy	
Driving Patient Care	• RP involved in decision making • RP involved in hands-on patient care • RP allowed to complete simple tasks independently
Taking the Wheel	• RP able to lead communication with family • RP able to handle day-to-day care responsibilities
Learner’s Permit	• Graduated level of responsibility • RP’s awareness of own limitations and knowing when to ask for help • AP providing safety net
Factors Promoting Resident Physician Autonomy	
Handing Over the Wheel	• Communication • Active promotion of RP decision making • Patient ownership • Team dynamics and hierarchy
Two Way Street	• Challenge RP to think independently • Remain open to RP input
Roadside Assistance	• Graduated independence • Allowing RP space to work • Providing opportunities for independent activity
Factors Undermining Resident Physician Autonomy	
Failing to Yield	• AP has predetermined course of action • Changing care plan without alerting or involving RP
Backseat Driving	• Micromanagement • Not leaving work area • Imposing personal treatment style

### Residents’ perception of what autonomy looks like?

*Driving Patient Care*: Residents as decision makers.

The need for graduated level of independence to assess a patient, select necessary investigations, develop a diagnosis, and formulate a treatment plan were often mentioned as critical components of the decision making process.

*So the more that you say, “This patient has this, I want to do this, this, and this,” […] [APs] at least have to respond to that plan. And I always am working toward trying to do that with every patient […] I think you can do things to buy yourself more autonomy.* (Emergency Medicine)

[As an example of autonomy…] *he was perfectly ok with me seeing the child, ordering the tests, coming with the results, getting the script ready, talking with the family about the course of the illness, and then he had to come in with me, to kind of give his blessing.* (Pediatrics)

*Taking the Wheel:* Promoting ownership of patient care.

The RP taking a visible role as the leader of the team during discussions with patients and families or other members of the health care team was another central element of autonomy.

*You can be as good as you want about writing notes and making decisions, but part of [autonomy] it is communicating to the patient …so [residents] need to get comfortable talking for their patients.* (Pediatrics)

*If you don’t get to enact it, then […] there’s no risk. So, if you don’t take the risk you don’t learn from the mistakes that you make or from the ramifications of those actions. I think it’s important to actually see the results of the decisions you make.* (Internal Medicine)

*Learner’s Permit:* Gaining autonomy with experience.

RPs recognized the importance of both adequate supervision to becoming efficient caregivers and of being afforded the appropriate level of graduated responsibility for patient care. They were aware of the distinction between “competence” and “training level,” and noted that these two were not necessarily directly connected.

*[…] in procedures, as the staff you’re working with becomes more comfortable with the resident, then they will provide you more and more leeway as far as performing the procedure more and more independently.* (Radiology)

*I just have the sense that [autonomy is] graduated, like raising a child. You’re going to slowly give them more decision-making power because you trust that they have the skills to make decisions.* (Psychiatry)

The importance of having the AP serve as a safety net in times of need was a common theme. Despite wishes to be autonomous, RPs understood the importance of maintaining patient safety. Additionally, RPs identified learning when to ask for help as an important developmental milestone on the path to becoming an independent physician.

[…] *a lot of times if you make the call and you say, “I need you to come in because I’m in over my head,” they don’t ask questions. They understand that you know [when you need assistance], and we’ve just built that relationship with them that they understand that*. (Pediatrics)

### Residents’ perception of what promotes learner autonomy?

*Handing Over the Wheel:* Learners leading patient care.

APs who promoted autonomy actively encouraged RPs to take the lead on patient care, emphasizing ownership of the patients in their care. Allowing time for and expecting RP to develop a diagnosis and treatment plan was perceived as having a positive impact on RP autonomy.

*I would tell them [APs] to have high expectations for us to first give our assessment [and] plan, and if they disagree with it then have an informed conversation about why they disagree with it or […] even if they agree with it, what we could do better.* (Internal Medicine)

*Some staff are very good […] about introducing themselves but saying […]“These are your doctors, they’re the ones making the decisions, I’m just here for assistance if they need it.”* (Internal Medicine)

Empowering the senior RP as a “middle man” between the junior RPs and APs reinforced the importance of the team dynamic and allowed RPs more autonomy in their daily activities.

*We’re trying to switch our family-centered rounds so they’re more senior resident driven rather than faculty driven. […] the med students and interns present to the senior resident and the senior resident tries to lead that discussion with the family, and then the attending comes in more as a supervisory role rather than as a team-leading type role.* (Pediatrics)

*Two Way Street*: Collaborative management of care.

APs who specifically asked what RPs want to do in given situations, their rationale, and discussed with the RP next steps in decision making and patient care were seen as reinforcing autonomy. APs who were receptive to the ideas proposed by the RP were identified as an important contributor to autonomy.

*If you have a critically ill patient, what really helps is that they actually stop when the decision is made and say, “Hey, what do you want to do now?” There’s sometimes, like with a coding patient where the really good attendings, even though something needs to be done fast, they’ll stop and say, “Hey, what do you want to do right now?”* (EM)

*Their answer’s never yes or no, it’s always, “Well why do you want to do that?” And some people think that’s annoying, but it’s not. That’s how you learn. Being challenged on, “Well why did you pick that medicine?” You tell them, and they’re like, “Ok, well how’s it going to act?” And you get to the end, and it’s like, “Yeah, you were right the whole time and I could have just said yes and moved on, but now you know why you did it.”* (Internal Medicine)

*And understanding both from the resident and the attending side that there’s a wide range of the “right way” to do things. There’s a lot of different ways to do it without being the “wrong way.” And as long as the resident isn’t doing it the wrong way, from our standpoint we feel like we’re being autonomous when we’re making those decisions.* (Emergency Medicine)

*Roadside Assistance:* Supervising from a distance.

A commonly cited perception of supervising appropriately was having the AP “disappear” for parts of the day.

*They basically said, “I’m going to go to my office, […] page me if you need me but this is your patient, you’re responsible for them. If you have questions, ask your senior, if you have any more questions call me.” I think it helps formulate plans better and makes you actually care more about patients, too, because you’re actually actively doing more with the patients.* (Psychiatry)

This point was reinforced by the RPs’ perception of on-call situations as being the times when they were able to operate most autonomously.

*When we’re on call there’s no staff in house. We fend for ourselves. They have a pager and we have access if we have questions. […] we’re set up to have our own decisions. If it’s a difficult case we can call them but that’s kind of the overriding way they allow us to make decisions on our own.* (Radiology)

### Residents’ perception of what undermines learner autonomy?

*Failing to Yield*: Minimizing resident involvement.

AP having a predetermined approach to patient care was perceived as preventing an active role in care for the RP. APs seeing patients before RPs had a chance to do initial workup and assessment, as well as placing orders (labs, imaging studies), performing procedures, or otherwise managing the delivery of care without them undermined autonomy. RPs also expressed frustration with APs who changed agreed upon plans, without notifying RPs.

*[…] there are some attendings that are like that, that it doesn’t matter so much what your plan is, their plan is going to be different most of the time. And that can be very frustrating, and that’s when you feel like you’re a technician and not a resident in training.* (Emergency Medicine)

*The time I have an issue is when they come to a plan of action, have you talk to the family, then change their mind shortly thereafter, and totally go over your head to change the plan mid-day while you’re out in the clinic or something. Then you find out later they were changed for totally arbitrary reasons. It just really makes you look terrible in front of the family.* (Internal Medicine)

*Backseat Driving:* Micromanaging patient care.

APs who exerted too much of an influence on minor decisions in patient care were perceived as depriving RPs of their ability to act independently.

*[…] if you’re going to micromanage us left and right, that really does hamper our knowledge, it hampers us from acquiring new information, it doesn’t let us feel out what we’re comfortable with doing because you’re making all the decisions.* (Internal Medicine)

APs who imposed personal style on patient care without providing an evidence-based reason for these decisions were felt to detract from RP learning and autonomy.

*I think people I respect most are those who – they don’t just tell you whether you’re wrong or right but they’ll give you the evidence behind it. Tell you why they’re choosing it, not just because it’s their personal preference […] if there’s an evidence-based reason for why they’re telling me I’m wrong, then I want to know about it.* (Internal Medicine)

Being interrupted while presenting their patients during rounds was perceived by RPs as interfering with their autonomy. When asked what one would see if watching for autonomous behavior during the day, some residents responded:

*See how many times the attending physicians interrupt you.* (Pediatrics)

*That’s disrespectful to a resident in general – some staff don’t even listen to you during rounds when you’re giving the numbers. Try to see if they’re paying attention.* (Pediatrics)

## Discusssion

This study across several specialties helps to construct a more cohesive understanding of RP perceptions of what autonomy looks like and how it is either promoted or undermined by AP activities. Table 4 summarizes key elements of autonomy as perceived by residents, which can be addressed by APs in daily practice. Promoting the RP’s ownership of their patients, allowing a leadership role, and encouraging active participation in clinical decision making can be accomplished through verbal acknowledgement of roles and careful discussions of clinical care. Perhaps most easily implemented is avoidance of interrupting the RP patient presentations, including the assessment and plan. Challenging residents to support their care plan while remaining supportive of their thought process is critical, as is basing any changes from RP plan on evidence rather than style. Being available at a distance as a “safety net” is important for the comfort level of RPs, but allowing time to work in the absence of the AP is also important for autonomous activity.

**Table 4:** Practical steps for promoting learner autonomy

What to do	What to avoid
• Clear communication regarding roles and responsibility • Encourage patient ownership • Actively promote RP decision making • Collaborate with RPs in decision making • Be sensitive to team dynamics and hierarchy • Allow case presentation without interruption • Challenge RP to think independently • Remain open to RP input • Provide evidence for alternative approaches • Graduated independence • Allow RP space to work • Provide opportunities for independent activity	• Having a predetermined course of action • Interrupting case presentations • Not asking RP for care plan and thought process • Changing care plans without RP involvement or knowledge • Constant presence in RP work area • Imposing personal management style

Some themes identified in this work reinforce previous published findings which were more limited in their scope of study in regard to diversity of research subjects or topical focus. RPs felt more autonomous when APs helped them to feel involved in the clinical decision making process and promoted ownership of patients, as previously seen in studies of IM and Pediatrics RPs [12, 18, 19]. The importance of taking the lead in communication with patients and other physicians and also of having an established hierarchy in seeking assistance was also highlighted in our study across different specialties [20, 21]. RPs in our study noted that a key component of developing autonomy is being provided appropriate supervision for their skill level. They did appreciate the difficulty APs experience in balancing the need for promoting autonomy while minimizing the frequency of provider error, and appeared to understand the differences in both achieving this balance and in how this balance is perceived differently by RPs and APs [12, 22]. It was also of interest that much of what the RPs shared related to daily rounds. While rounding is only a small portion of daily activities on the wards, it is possible that this sets the stage for an RP’s sense of autonomy throughout the remainder of the day. Regardless, the importance of fostering autonomy in all daily activities must be considered by clinical educators.

Taken together, these results shed light on new elements important to RPs’ perception of autonomy and reinforce the importance of previously identified elements, as well as providing evidence to their generalizability across specialties. We realize that the development of trust between APs and RPs is a critical step which allows increased responsibility and the provision of autonomous decision making. Development of this trust not only depends on competence (knowledge or skills) but is complemented by integrity (truthfulness and benevolence), reliability (predictable behavior) and humility (insight into own limitations and willingness to ask for help if needed) [23]. Graduated level of independence and entrustment addresses the tension between supervision and autonomy by appropriately increasing independence without affecting patient safety [6]. Identifying those factors which contribute most to RPs’ perceptions of their own autonomy will allow APs to alter their approach to learners in order to best support their development as independent practitioners.

There were limitations to this study. Participants were only from Iowa residency programs and RP experiences in other states, particularly those with very different patient populations, may be quite different, resulting in varied thoughts on the concept of autonomy. RPs in more procedure-heavy fields (surgery, OB-GYN, anesthesia, etc.) were not included in this study, and future research is needed to determine if they have different perceptions of these issues. The limited number of residency programs that participated, and particularly limited numbers of resident participants within each of the group meetings, did not allow for meaningful analysis of differences in RP perspectives between specialty and/or residency program. However, the number of programs and participants was appropriate for an exploratory study of this type seeking to identify the range of perspectives that residents have in regard to this issue. The multimethod approach used to data collecting, combining open ended survey questions with facilitated group discussion, allowed for a maximum range of perspectives to be gathered and analyzed. It would be advantageous to expand this study to other programs at different institutions to see if the range of responses and main themes identified in this study are more generalizable would allow identification of differences in perceptions and experiences of residents at different levels and in different disciplines. Finally, this study did not examine the views of APs. Previous work has shown that there is often a disconnect between RPs and APs regarding autonomous behavior and abilities, and that even within groups of APs there can be different perceptions of autonomy based on experience level, the field in which an AP/RP pair practice, and other characteristics that may vary between physicians [1, 12, 22, 24, 25]. Determining how best to discern when to entrust specific tasks to RPs is an ongoing point of discussion [1, 23, 26].

This study provides insights into RP perceptions of APs behaviors that either promote or undermine learner autonomy. The persistence of the themes identified in this work through several different residency programs suggest that they are not unique to any one learner or discipline, but rather represent concerns that may be generalized to RPs as a whole. Future research should explore effective methods of adapting graduate medical education to address these concerns without sacrificing quality patient care. The driving metaphors used throughout this study arose from language used by the learners themselves throughout our discussions. The use of such language seemed to us quite fitting as, just as with learning to drive, if a person is never given a chance to sit behind the wheel independently, they will never be able to perform without assistance from the instructor. Through this work we have provided a road map whereby APs can cede the wheel to learners while still assuring all parties safely arrive at their destination.

## Conclusions

Fostering autonomy is a critical aspect of medical education. There are several steps attending physicians can take to support this autonomy, including clearly stating roles, encouraging patient ownership, including residents in clinical decision making, and providing graduated levels of independence. Residents understood the challenges their supervisors face in providing autonomy, and appreciated even small steps taken to provide more independence.

## Data Availability

The datasets generated during and/or analyzed were audio recorded and are not publicly available due to personal nature of some of the information included, but are available from the corresponding authors on reasonable request.

## Abbreviations

RP: Resident Physician(s)
AP: Attending Physician(s)
CBME: Competency Based Medical Education
EPA: Entrustable Professional Activities
